# Experimental Study on the Preparation of High-Purity Iron Oxide Red by Acid Leaching Iron from Coal Gangue

**DOI:** 10.3390/ma17133275

**Published:** 2024-07-03

**Authors:** Xulong Yang, Aiyuan Ma, Ming Chen, Jinsong Du, Xuemei Zheng

**Affiliations:** 1 College of Environmental and Chemical Engineering, Dalian University, Dalian 116622, China; m13203976597@163.com (X.Y.); 13733025795@163.com (J.D.); 2 School of Chemistry and Materials Engineering, Liupanshui Normol University, Liupanshui 553004, China; 18183439860@163.com (M.C.); zxm_lpssy19@163.com (X.Z.)

**Keywords:** coal gangue, roasting activation, acid leaching, solvent extraction method, iron oxide red

## Abstract

Aiming at the problems of the large storage, complex composition, low comprehensive utilization rate, and high environmental impact of coal gangue, this paper carried out experimental research on the preparation of iron oxide red from high-iron gangue by calcination activation, acid leaching, extraction, and the hydrothermal synthesis of coal gangue. The experimental results show that when the calcination temperature of coal gangue is 500 °C, the calcination time is 1.5 h, the optimal concentration of iron removal is 6 mol/L, the acid leaching temperature is 80 °C, the acid leaching time is 1 h, and the liquid——solid mass ratio is 4:1; the iron dissolution rate can reach 87.64%. A solvent extraction method (TBP-SK–hydrochloric acid system) was used to extract the leachate, and a solution with iron content up to 99.21% was obtained. By controlling the optimum hydrothermal conditions (pH = 9, temperature 170 °C, reaction time 5 h), high-purity iron oxide red product can be prepared; the yield is 80.07%. The red iron oxide was characterized by XRD, SEM-EDS, particle-size analysis, and ICP-OES. The results show that the red iron oxide peak has a cubic microstructure, an average particle size of 167.16 μm, and a purity of 99.16%. The quality of the prepared iron oxide red product meets the requirement of 98.5% of the “YHT4 Iron oxide Standard for ferrite”. It can be used as a raw material to produce high-performance soft magnetic ferrite. In summary, this experimental study on the preparation of iron oxide red from coal gangue is of great significance for the comprehensive utilization of coal gangue to realize the sustainable development of the environment and economy.

## 1. Introduction

**Figure 1:**
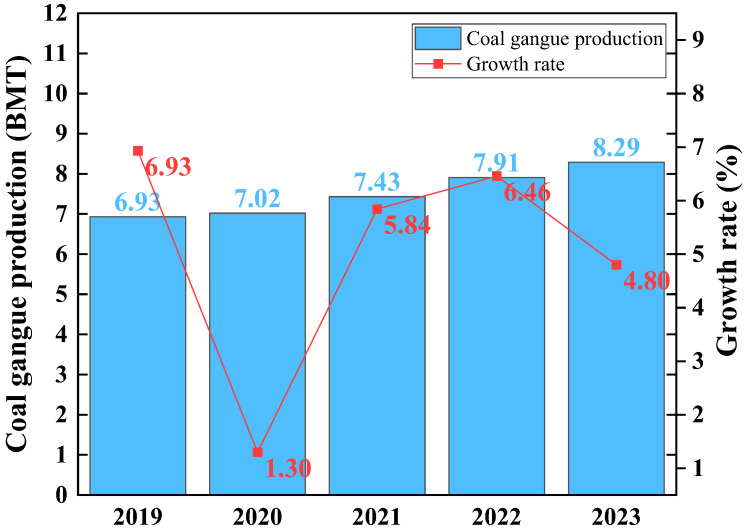
China’s annual coal gangue production and growth rate from 2019 to 2023.

Coal gangue is industrial waste generated during the mining and washing processes of coal, accounting for about 10–20% of coal production. Globally, coal gangue production is significant [1]. The large amount of coal gangue seriously harms the ecological environment and human health during transportation, stacking, and processing [2]. The annual output and growth rate of coal gangue in China from 2019 to 2023 are shown in Figure 1.The resource utilization of coal gangue in power generation [3], gas production [4], cement preparation [5], construction materials [6], and refractory materials [7] can reduce ecological environmental damage and increase industrial added value. However, it cannot achieve the high-value utilization of coal gangue. Therefore, exploring new pathways for preparing high-performance materials from coal gangue and improving its utilization efficiency is imperative [8].

Coal gangue contains a large amount of Si, Al, and Fe elements, mainly in the form of minerals such as kaolinite, quartz, and hematite, making it a potential mineral resource [9]. According to the aluminum–silicon ratio (Al_2_O_3_:SiO_2_) in coal gangue, it can be divided into three categories: aluminum–silicon ratio ≥0.5, aluminum–silicon ratio between 0.3 and 0.5, and aluminum–silicon ratio ≤0.3. Regarding coal gangue with an aluminum–silicon ratio ≥0.5, it has a high aluminum content, relatively low silicon content, and characteristics such as a small particle size and good plasticity, making it widely used in the preparation of zeolite molecular sieves. Wang et al. [10] used coal gangue as a raw material and employed an impregnation–calcination method to prepare coal gangue molecular sieve Cu/Fe-X catalysts, which were then used for removing Rhodamine B from aqueous solutions. Under optimal conditions, the catalytic degradation rate reached 99.9%, with a high TOC removal rate of 98.5%. Zheng et al. [11] utilized coal gangue as a silicon and aluminum source to successfully synthesize ZSM-5 molecular sieves. The sample exhibited a microporous specific surface area of 169.6329 m^2^/g, an average pore size of 0.6285 nm, and a pore volume of 0.0988 cm^3^/g. For coal gangue with an aluminum–silicon ratio between 0.3 and 0.5, the moderate aluminum and silicon content allows for the extraction of Al and Si elements for the production of various products, such as Al_2_O_3_ [12], AlCl_3_ [13], Na_2_SiO_3_, and SiO_2_ [14,15,16]. Han et al. [17] activated coal gangue using alkaline supercritical water, revealing extraction rates of Al^3+^ and Si^4+^ as high as 78.9% and 69.2%, respectively, after activation. Al_2_O_3_ and SiO_2_ were prepared using an acid-base combination method, with purities of 99.3% and 96.2%, respectively. Wang et al. [18] used a process of hematite roasting–alkaline leaching–Bayer process to extract Al_2_O_3_ and SiO_2_ from high-alumina coal gangue. At an alkaline leaching temperature of 110 °C and a Bayer digestion temperature of 260 °C, they obtained 96% SiO_2_ and 97% Al_2_O_3_, respectively. For coal gangue with an aluminum–silicon ratio ≤0.3, the silicon content is much higher than the aluminum content. With a large particle size and poor plasticity, it can be used to produce cement, ceramic tiles, and split bricks [19].

Additionally, coal gangue can be classified into low-iron coal gangue (0.1–1.0 wt.%), medium-iron coal gangue (1.0–8.0 wt.%), high-iron coal gangue (8.0–18.0 wt.%), and ultra-high-iron coal gangue (>18.0 wt.%) based on its iron content. High-iron and ultra-high-iron coal gangue have a higher iron content, which can be utilized for the production of Fe_2_O_3_. Iron elements in coal gangue primarily exist in the form of siderite and hematite, with relatively low grades [20]. Conventional physical beneficiation techniques encounter challenges in separating and enriching iron minerals, resulting in low economic efficiency. Currently, iron extraction mainly relies on acid leaching, a process involving three stages: acid leaching, filtration, and purification [21]. Zheng et al. [22] employed a response surface experimental design to optimize the preparation process of iron oxide red pigment from coal gangue aluminum–iron separation liquid. The results revealed that under conditions of a Na_2_CO_3_ concentration of 107 g/L, reaction temperature of 43 °C, and Fe^2+^ concentration of 0.26 mol/L, the purity of the obtained iron oxide red product could reach 96.09%. Kong et al. [23] conducted research on extracting iron ions from coal gangue. When coal gangue was roasted at 675 °C for 1 h and leached with 6 mol/L hydrochloric acid at 93 °C for 4 h, the leaching rate of iron ions exceeded 90%. However, in the process of preparing iron oxide red in the aforementioned studies, iron ions were precipitated by adjusting the pH value, resulting in the precipitation of other metal elements (such as Al and Ca) from the leach solution. This method faces challenges in controlling the pH value and can lead to the precipitation of certain impurities, thereby affecting the purity of the iron oxide red. Solvent extraction, characterized by its high selectivity, good economic efficiency, and feasibility for continuous operation, has been proven to be highly effective in metal recovery from various solutions [24]. Zhang et al. [25] investigated the selective extraction of Fe^3+^ from high-acidity chloride leach solution using a mixed solvent extraction agent composed of tributyl phosphate (TBP) and methyl isobutyl ketone (MIBK). They found that TBP and MIBK exhibited a synergistic effect in extracting iron ions, achieving a Fe^3+^ extraction rate of 91.0% under the conditions of a 70% TBP volume fraction, 2 min of contact time, and 25 °C temperature. Duan et al. [26] added P_2_O_4_ to the traditional TBP-FeCl_3_–kerosene system to selectively extract lithium from high magnesium/lithium ratio brine, thereby solving the problem of Fe^3+^ loss and improving the stability of the system. The single-stage extraction rate of Li^+^ was 52.71%. Using the solvent extraction method (TBP-SK-HCl system), Fe ions in the leaching solution are extracted and back-extracted multiple times to obtain a high-purity FeCl_3_ solution. The main advantages of this process are as follows: (1) a short extraction time and high extraction efficiency; (2) the low cost of TBP and sulfonated kerosene (SK), recyclable with high selectivity [27]; and (3) no introduction of alkali or other precipitants throughout the entire process, greatly enhancing the purity of the ferric oxide red.

To achieve the comprehensive utilization of coal gangue, this study utilizes high-iron coal gangue as the raw material to conduct experimental research on the preparation of high-purity ferric oxide red through the processes of coal gangue calcination activation, acid leaching iron removal, extraction, and hydrothermal synthesis. The experiment investigates four process parameter conditions (calcination activation, acid leaching iron removal, extraction purification, and hydrothermal synthesis), to prepare high-purity ferric oxide red products. The properties of ferric oxide red are analyzed using characterization methods such as XRD, SEM-EDS, particle-size analysis, and ICP-OES.

## 2. Materials and Methods

### 2.1. Materials

The raw materials for the experiment were sourced from a coal-mining enterprise in Liupanshui, China. The elemental composition of the coal gangue was determined using a Japanese Rigaku Supermini 200 wavelength-dispersive X-ray fluorescence spectrometer (XRF, Rigaku Company, Tokyo, Japan), as shown in Table 1. The chemical composition of the coal gangue is quite complex, containing a large amount of Si, Fe, Al, Ca, and other elements. Specifically, the SiO_2_ content is 35.5%, Fe_2_O_3_ content is 25.1%, and Al_2_O_3_ content is 14.7%, indicating it as a typical high-iron–low-aluminum coal gangue. To further determine the Fe content in the coal gangue sample, an Agilent 5800 inductively coupled plasma emission spectrometer (ICP-OES, Agilent Technologies Inc, Santa Clara, CA, USA) was used for measurements, revealing an Fe element content of 12.93% in the coal gangue.

**Figure 2:**
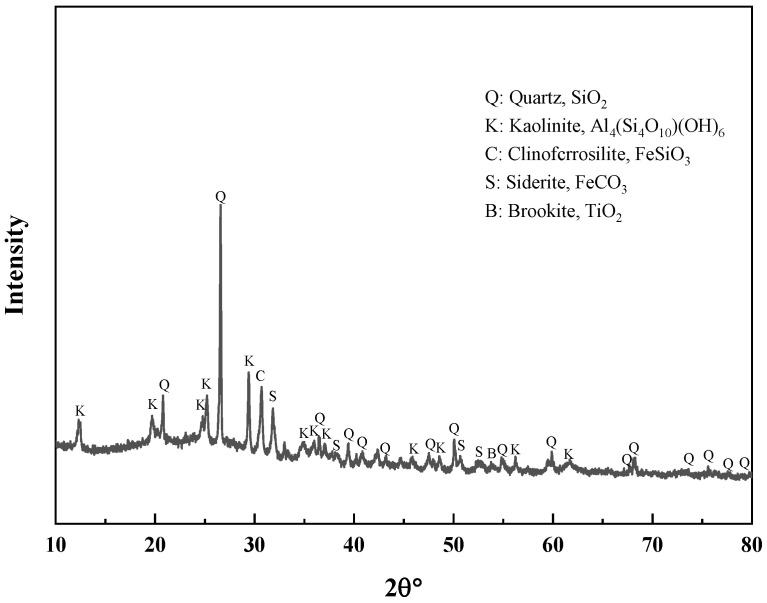
XRD pattern of sample.

**Figure 3:**
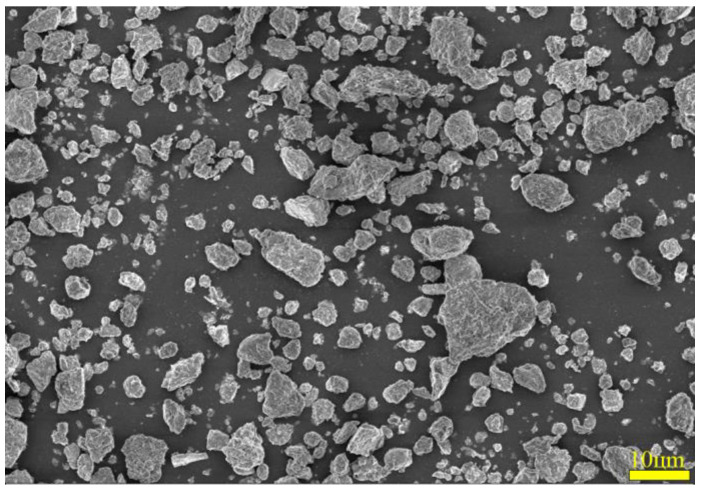
SEM pattern of sample.

**Figure 4:**
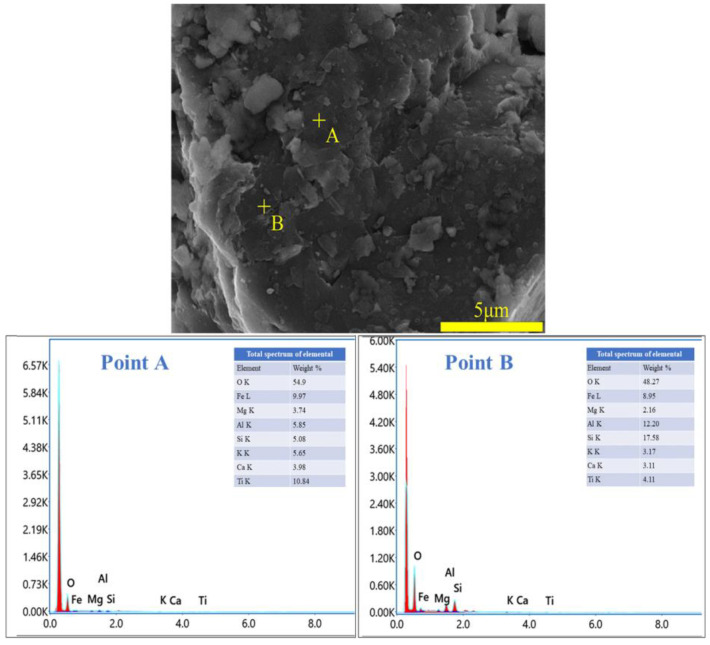
SEM image and EDS point analysis of sample.

**Figure 5:**
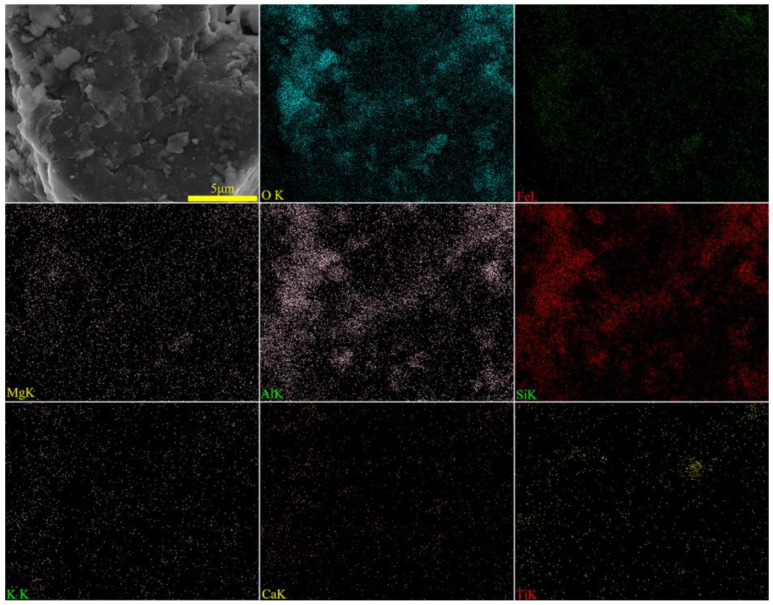
SEM image and EDS mapping analysis of sample.

To ascertain the mineral composition and structural morphology of the coal gangue, a Japanese Rigaku Ultima IV X-ray diffractometer (XRD, Rigaku Company, Tokyo, Japan) and a German ZEISS Sigma 300 scanning electron microscope (FE-SEM, Carl Zeiss AG, Oberkochen, Germany) were used to conduct XRD, SEM, and SEM-EDS characterization analyses of the coal gangue samples. The XRD, SEM, and SEM-EDS spectra of the coal gangue samples are shown in Figure 2, Figure 3, Figure 4 and Figure 5, respectively. The XRD results indicate that the main mineral components of the coal gangue are quartz (SiO_2_) and kaolinite (Al_4_(Si_4_O_10_)(OH)_6_). The diffraction peaks of the quartz phase are sharp and clear, suggesting high crystallinity. Additionally, the coal gangue contains small amounts of clinoferrosilite (FeSiO_3_), siderite (FeCO_3_), and brookite (TiO_2_). The SEM results showed that the size distribution of coal gangue particles is uneven and shows an irregular morphology. The SEM-EDS point and area scan analyses of the coal gangue raw material are shown in Figure 4 and Figure 5, respectively. From Figure 4, it is evident that the coal gangue particles are formed by stacking lamellar crystals, exhibiting a typical layered structure. The Fe element is uniformly distributed within the coal gangue particles. The coal gangue is primarily composed of elements such as Si, Fe, Al, Ca, Mg, S, K, among others, which corresponds well with the analysis results in Table 1. From Figure 5, it can be observed that the combination and dispersion of O, Fe, Al, and Si are relatively uniform, consistent with the phase analysis results in Figure 2. The coal gangue is mainly composed of phases such as SiO_2_, Al_4_(Si_4_O_10_)(OH)_6_, and FeSiO_3_.

### 2.2. Methods

#### 2.2.1. Acid Leaching Experiments

The coal gangue is subjected to drying, crushing, and grinding treatments. A certain mass of coal gangue is placed in a muffle furnace and roasted at different temperatures (300 °C, 400 °C, 500 °C, 600 °C, 700 °C) and for different roasting times (0.5 h, 1.0 h, 1.5 h, 2.0 h, 2.5 h), then allowed to cool naturally before retrieval. Using deionized water to dilute the concentrated hydrochloric acid, we prepare hydrochloric acid solutions of different molar concentrations (1 mol/L, 3 mol/L, 4 mol/L, 6 mol/L, 8 mol/L). A total of 10 g of pre-roasted coal gangue samples is taken and placed in conical flasks with hydrochloric acid solutions of different molar concentrations. The heating, stirring, and leaching is performed under different reaction temperature (50 °C, 60 °C, 70 °C, 80 °C, 90 °C), reaction time (0.5 h, 1.0 h, 1.5 h, 2.0 h, 2.5 h), and liquid——solid mass ratio (2:1, 3:1, 4:1, 5:1, 6:1) conditions. After leaching, the mixture is filtered to obtain the leachate and filter residue. The Fe content in the leachate is determined by the ortho-phenanthroline colorimetric method using a TU-1901 UV–visible spectrophotometer (UV–Vis, Beijing Purkinje GENERAL Instrument Co., Ltd., Beijing, China), while the mass fraction of Fe in the coal gangue raw material is determined by ICP-OES. The leaching rate of Fe M% is calculated by the following formula:

(1) $$M=\frac{cnV}{m\omega }\times 100\%$$

where *c* (mg/L) represents the Fe concentration determined by UV–Vis spectrophotometer; *n* indicates the multiple of the dilution of the leachate; *V* (L) indicates the total volume of the leachate; m (g) indicates the mass of coal gangue; and ω (%) indicates the mass fraction of Fe in the coal gangue.

#### 2.2.2. Extraction Experiments of Leachates

According to the optimal technological parameters, the coal gangue is calcined and leached, and multiple leaching solutions are collected and heated to concentrate them. The specific process is as follows: Five groups of leaching solutions are collected and put into beakers, heated to boiling on an electric stove, and concentrated to 500 mL. Then, the concentrated solution is used for subsequent extraction experiments. In the extraction process, Fe^2+^ in the percolate will be oxidized to Fe^3+^. After cooling, 12 mol/L HCl is added to increase the acid concentration of the solution to above 4 mol/L. Use tributyl phosphate as the extractant and sulfonated kerosene as the diluent. Extract Fe from the leachate under the conditions of 40% TBP concentration, O/A = 2:1 (organic phase/aqueous phase), an extraction temperature of 20 °C, and extraction time of 10 min. Strip the Fe using deionized water as the stripping agent under the conditions of O/A = 1:1, a stripping temperature of 20 °C, and stripping time of 10 min. Repeat the extraction–stripping process 2–3 times (TBP extract can be reused during the process). Finally, obtain the coal gangue-stripping solution containing high-purity Fe ions.

Use the Agilent 5800 inductively coupled plasma emission spectrometer to detect the ion concentrations of Fe, Al, Ca, and Mg in the leachate after extraction and in the back-extraction solution. The calculation formula is as follows:

(2) $$C_{x}=\frac{C_{0}\times f\times V_{0}\times 10^{-3}}{m_{0}\times 10^{-3}}=\frac{C_{1}\times V_{0}\times 10^{-3}}{m_{0}\times 10^{-3}}$$

C_0_: the concentration of the test solution elements, in mg/L; *f*: the dilution factor; *V*_0_: the volume after the sample is digested and made up to volume, in mL; *m*_0_: the mass of the coal gangue raw material taken, in grams; C_1_: the elemental concentration of the sample digestion solution, in mg/L; C_x_: the concentration of the measured element, in mg/L.

#### 2.2.3. Preparation of Iron Oxide Red

**Figure 6:**
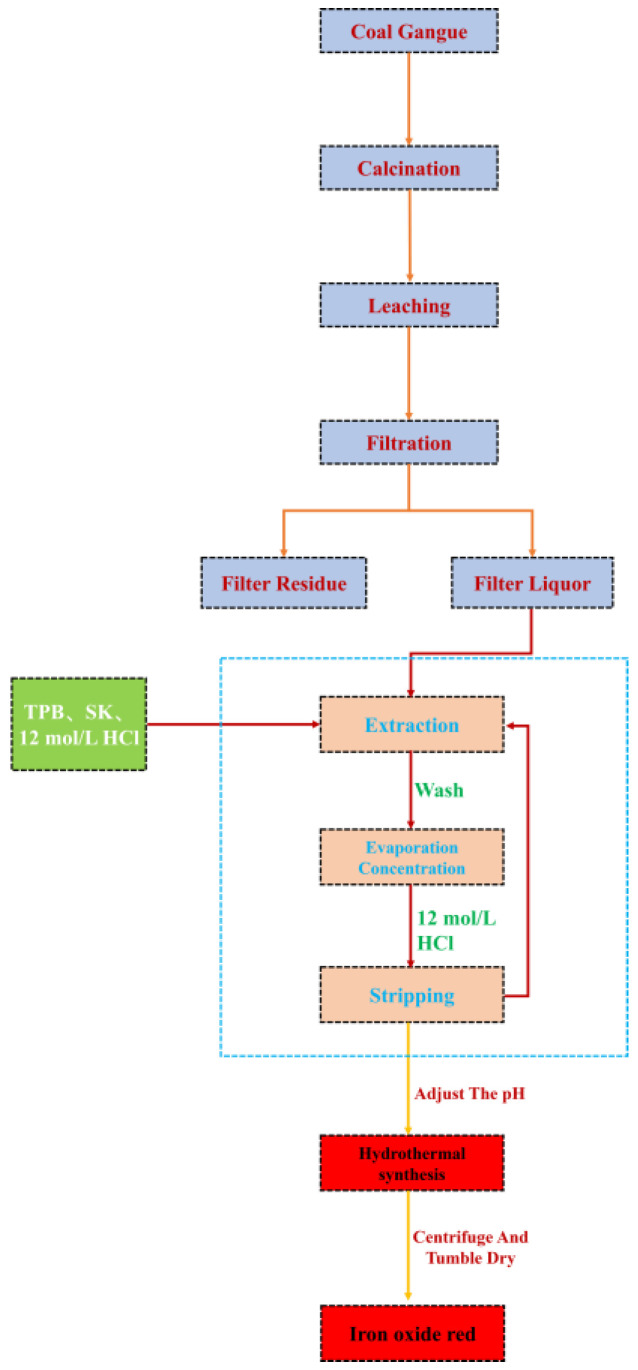
Process flowchart of iron red prepared by iron liquid extraction by coal gangue acid leaching.

The extracted solution is transferred to a beaker, stirred at 60 °C, and then 2 mol/L NaOH solution is added to adjust the pH. The mixture is stirred for 1 h at 80 °C, then transferred to a hydrothermal reaction vessel. Under different hydrothermal temperature, time, and pH conditions, hydrothermal reactions are carried out. Finally, the solution is centrifuged, dried, and ground to obtain Fe_2_O_3_. The process flowchart for preparing ferric oxide from coal gangue is shown in Figure 6.

## 3. Results and Discussion

### 3.1. Experimental Study on Coal Gangue Roasting Process

**Figure 7:**
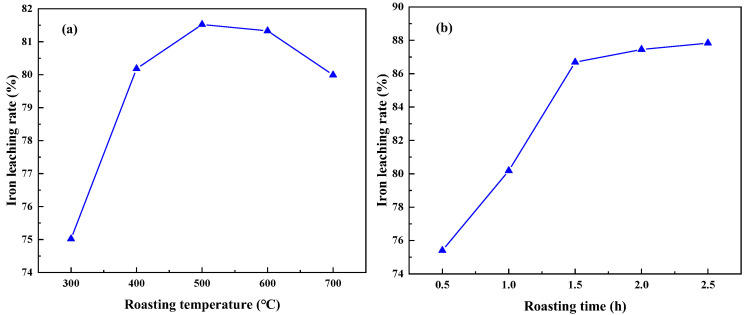
Effect of different roasting temperature (**a**) and roasting time (**b**) on iron leaching rate (acid leaching temperature of 70 °C, leaching time of 2 h, liquid—solid mass ratio of 4:1, and acid concentration of 6 mol/L).

The experiment was conducted under the conditions of an acid leaching temperature of 70 °C, leaching time of 2 h, liquid——solid mass ratio of 4:1, and acid concentration of 6 mol/L. The effects of different roasting temperatures and roasting times on the iron removal rate in coal gangue were investigated. The results are shown in Figure 7. Figure 7a illustrates that as the roasting temperature increases, the iron removal rate gradually increases. When the roasting temperature reaches 500 °C, the highest iron removal rate is 81.52%. At this temperature, the coal gangue exhibits the highest reaction activity and maximum specific surface area, which is conducive to the leaching of Fe_2_O_3_. However, a further increase in temperature leads to a decrease in the specific surface area of the coal gangue and a reduction in activity, resulting in a decrease in the iron removal rate [28]. Therefore, the optimal roasting temperature is determined to be 500 °C. Figure 7b shows that with an increase in roasting time, the iron removal rate gradually increases. After a roasting time exceeding 1.5 h, the change in the iron removal rate is insignificant. Therefore, the optimal roasting time is determined to be 1.5 h.

**Figure 8:**
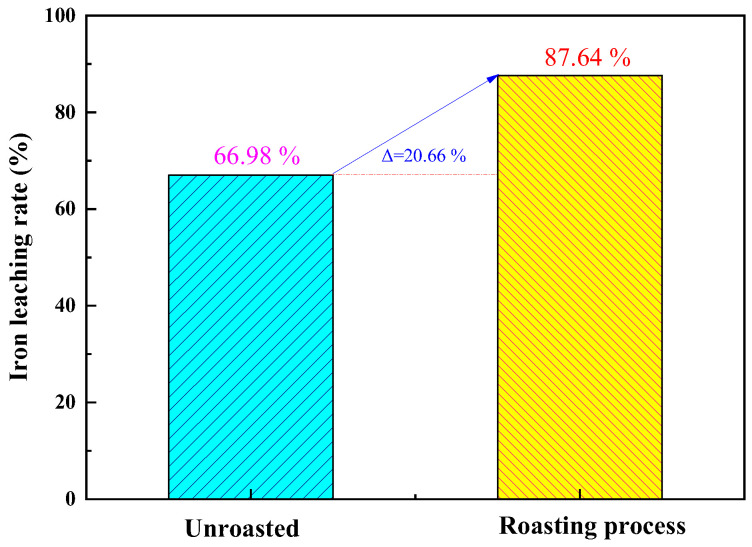
Comparison of iron leaching rate between unroasted and roasting-activated treatments of coal gangue.

Under optimized roasting activation conditions, a comparative study was conducted to investigate the effects of unroasted and roasted activation on the iron removal rate of coal gangue. The results, as shown in Figure 8, indicate that the iron removal rate of unroasted coal gangue is 66.98%. Unroasted coal gangue exhibits a higher lattice structure and lower reaction activity. The iron removal rate of roasted activated coal gangue is 87.64%. Under high-temperature roasting, particles undergo intense thermal motion, causing ions such as Fe, Mg, and Ca to reselect filling positions and leading to the formation of short-chain, disordered, and unstable glassy and amorphous substances from silicon–oxygen tetrahedra and aluminum–oxygen octahedra. This greatly enhances the reaction activity [29]. Comparative experimental results demonstrate that the roasting activation of coal gangue contributes to an increased leaching rate of Fe.

### 3.2. Experimental Study on Coal Gangue Roasting–Acid Leaching Iron Removal Process

#### 3.2.1. The Influence of Different Leaching Conditions on Iron Leaching Rate

**Figure 9:**
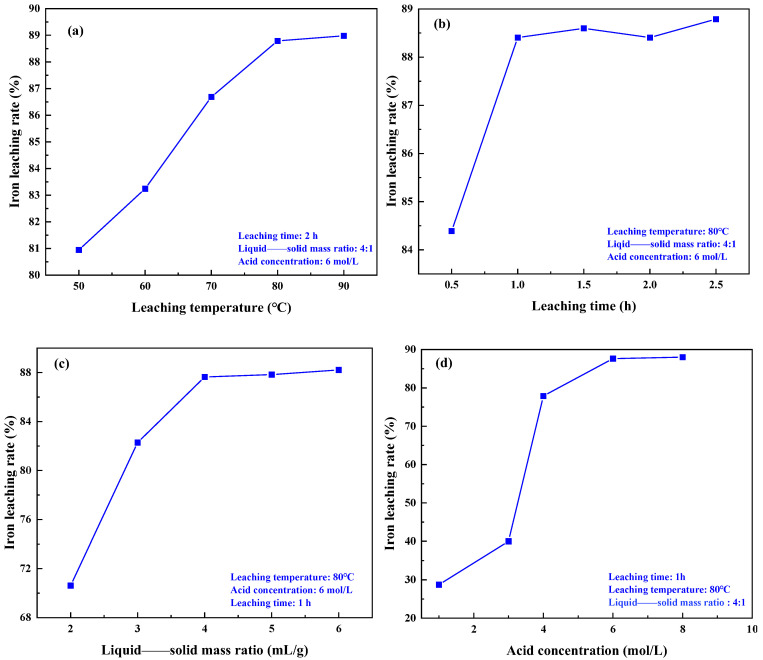
Effect of different leaching conditions on iron leaching rate: (**a**) temperature, (**b**) time, (**c**) liquid——solid mass ratio, (**d**) acid concentration.

The experiment investigated the effects of different leaching temperatures, leaching times, liquid——solid mass ratios, and acid concentrations on the iron removal rate in coal gangue. The results are depicted in Figure 9. Figure 9a demonstrates that with an increase in leaching temperature, the iron removal rate gradually rises. Higher temperatures enhance the material activity within the coal gangue, promoting acid leaching reactions [30]. However, when the leaching temperature is increased from 80 °C to 90 °C, the iron removal rate remains almost unchanged. This mainly causes the evaporation of the solution water due to the high temperature, which accelerates the evaporation of the solution and increases the concentration of hydrochloric acid, which leads to a reduction in the diffusion of HCl and the surface of the gangue particles, thus hindering the leaching of Fe ions. Consequently, the optimal leaching temperature is determined to be 80 °C. The results in Figure 9b indicate that as the acid leaching time increases, the iron removal rate gradually rises. After 1.0 h of acid leaching, the iron removal rate increases slowly. Therefore, the optimal leaching time is determined to be 1.0 h. The results in Figure 9c demonstrate that at lower liquid——solid mass ratios, the iron removal rate increases with an increase in the liquid——solid mass ratio. This is because at lower liquid——solid mass ratios, the hydrochloric acid content is lower, resulting in an incomplete reaction between the iron oxide in the coal gangue and the hydrochloric acid, leading to a lower leaching rate of Fe ions. As the liquid——solid mass ratio gradually increases, the hydrochloric acid content increases, leading to a more complete reaction and promoting the leaching of Fe ions. When the liquid——solid mass ratio reaches 4:1, the reaction between the coal gangue and hydrochloric acid is sufficient, and the iron removal rate mainly depends on the temperature. As the liquid——solid mass ratio continues to increase, the iron removal rate remains relatively unchanged. Therefore, the optimal liquid——solid mass ratio is chosen to be 4:1. Figure 9d illustrates that as the hydrochloric acid concentration increases, the iron removal rate continues to rise. This is because, with the increase in hydrochloric acid concentration, the probability of H^+^ contacting iron bearing minerals increases, thereby enhancing the reaction rate. However, when the acid concentration exceeds 6 mol/L, the iron removal rate does not increase significantly. Moreover, an excessively high hydrochloric acid concentration exacerbates side reactions, which is detrimental to the subsequent wastewater treatment and deep processing of the coal gangue. Therefore, the optimal acid concentration was selected as 6 mol/L. In summary, the optimal conditions for each factor are a leaching temperature of 80 °C, an acid leaching time of 1 h, a liquid——solid mass ratio of 4:1, and an acid concentration of 6 mol/L, resulting in an iron removal rate of 87.64%.

#### 3.2.2. Coal Gangue Leaching Residue XRF, XRD, SEM-EDS, and Particle-Size Distribution Characterization Analysis

Table 2 shows the chemical composition of the coal gangue leaching slag, and by comparing it with Table 1, it is found that the Fe_2_O_3_ components in the coal gangue after acid leaching are basically removed and separated, and the contents of CaO and MgO are also greatly reduced, while the SiO_2_ and most of the Al_2_O_3_ components are retained, and the total mass of the two components reaches 78.2%. It can be seen that the grade of the acid leaching of the coal gangue has been effectively improved through heat activation.

**Figure 10:**
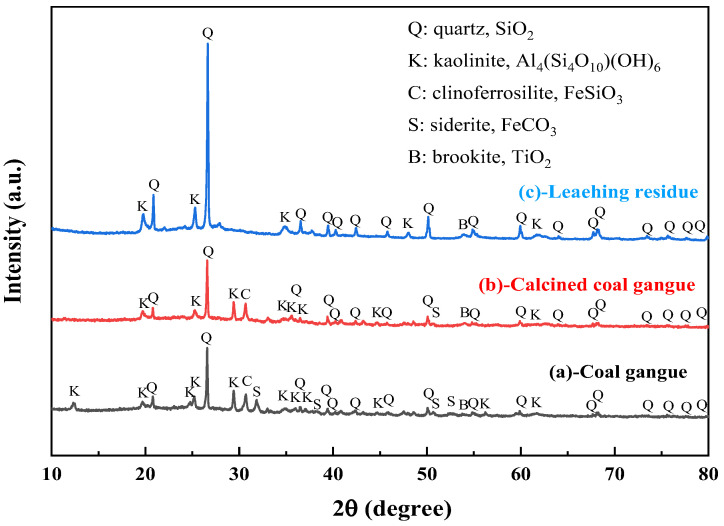
XRD patterns of coal gangue raw materials (**a**), calcined coal gangue (**b**) and leaching residue (**c**).

**Figure 11:**
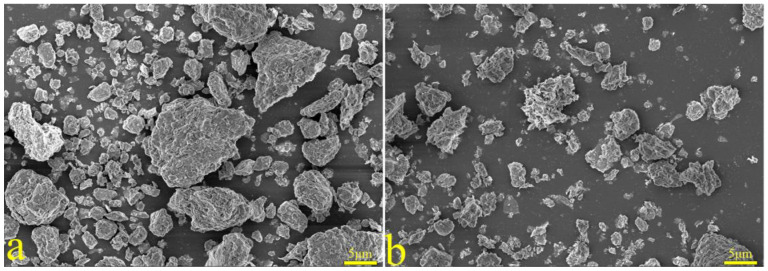
SEM patterns of calcined coal gangue (**a**) and leaching residue (**b**).

**Figure 12:**
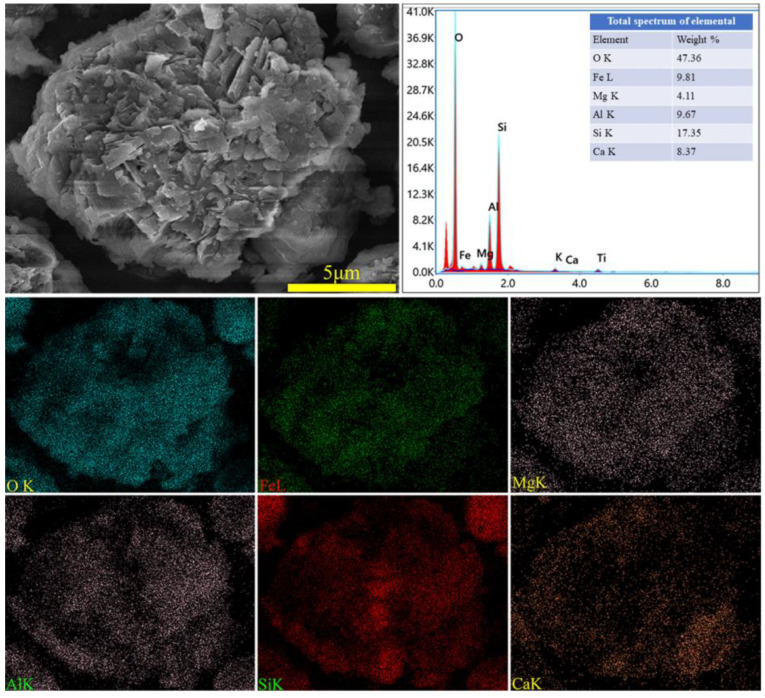
SEM image and EDS mapping analysis of calcined coal gangue.

**Figure 13:**
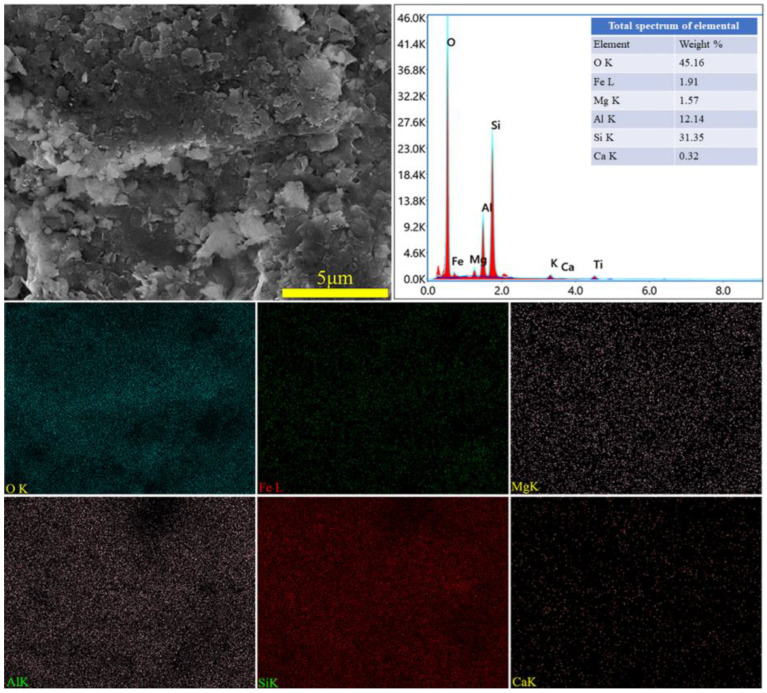
SEM image and EDS mapping analysis of leaching residue.

**Figure 14:**
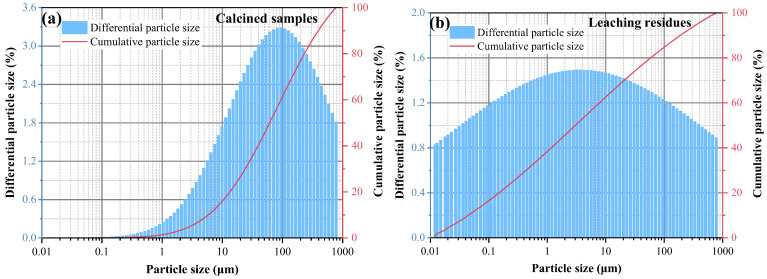
Particle-size distribution diagrams of coal gangue (**a**) calcined samples and (**b**) leaching residues.

The XRD spectra of the gangue feedstock, calcined samples, and leaching residue are shown in Figure 10, which shows that the diffraction peak of siderite disappears after calcination, and siderite is oxidized at high temperatures to form Fe_2_O_3_ [31]. The XRD pattern of the leaching residue lacks the diffraction peaks associated with siderite and orthopyroxene phases, indicating their reaction with hydrochloric acid to produce Fe ions and SiO_2_. Additionally, the diffraction pattern of the leaching residue is similar to that before acid leaching, but with different intensities. This is primarily because quartz and brookite do not react with hydrochloric acid. The dissolution of Fe and Al ions after acid leaching increases the relative content of the remaining substances, resulting in an enhanced diffraction intensity. The SEM micrographs of the calcined sample and the leaching residue of the coal gangue are shown in Figure 11. From the images, it can be observed that the calcined sample contains many large particles, ranging from 15 to 20 μm or even larger. However, after acid leaching, the particle size decreases, with most particles having diameters smaller than 5 μm [32]. This reduction in particle size is attributed to the leaching of Fe and Al ions, which leads to the loosening of residual particles, resulting in the presence of numerous fragments. The SEM-EDS spectrum of the calcined sample is shown in Figure 12. Compared to the raw material, the surface of the calcined sample particles becomes porous and loose. This is mainly attributed to the reaction of certain components of the coal gangue (such as kaolinite and siderite) at high temperatures, leading to the formation of numerous pores within the coal gangue, thus resulting in a porous and loose structure. This structure provides favorable conditions for subsequent acid leaching processes, enhancing the leaching of Fe ions. The iron (Fe) content in the calcined sample is 9.81 wt%, with the elemental content ranking as follows: O (47.36 wt%) > Si (17.35 wt%) > Fe (9.81 wt%) > Al (9.67 wt%) > Ca (8.37 wt%) > Mg (4.11 wt%). The SEM-EDS spectrum of the leaching residue is shown in Figure 13. Compared to the calcined sample, the surface of the leaching residue exhibits obvious signs of erosion, indicating that hydrochloric acid has dissolved some elements from the coal gangue and entered the leachate. Additionally, the iron (Fe) content in the leaching residue is only 1.91 wt%, with the elemental content ranking as follows: O (45.16 wt%) > Si (31.35 wt%) > Al (12.14 wt%) > Fe (1.91 wt%) > Mg (1.57 wt%) > Ca (0.32 wt%). This suggests that most of the iron in the coal gangue has been leached out through the acid leaching process, which is consistent with the XRD analysis results. The particle size analysis of the calcined sample and leaching residue was conducted using a Winner2005A laser particle size analyzer (LPS, JINAN WINNER PARTICLE INSTRUMENT STOCK CO., LTD., Jinan, China), with the results shown in Figure 14. The particle-size distribution of the calcined samples is shown in Figure 14a and ranges from 0.01 to 780 μm, with an average particle size of 61.64 μm. The cumulative volume of particles smaller than 100 μm is 84.19%, and for particles smaller than 500 μm, the cumulative volume is 96.30%. The volume-specific surface area is 0.557 m^2^/cm^3^. The particle-size distribution of the leaching residue is shown in Figure 14b and ranges from 0.01 to 780 μm, with an average particle size of 3.045 μm. The cumulative volume of particles smaller than 100 μm is 59.90%, and for particles smaller than 500 μm, the cumulative volume is 91.92%. The volume-specific surface area is 41.33 m^2^/cm^3^. After acid leaching, the overall particle size of the coal gangue decreased significantly, which was consistent with the characterization results of the SEM. This phenomenon may be caused by the destruction and dissolution of the activated components in the coal gangue.

### 3.3. Experimental Study on Preparation of Iron Oxide Red from Iron-Bearing Leachate of Coal Gangue

The leachate obtained by filtering the acid-leached samples of coal gangue after roasting activation was subjected to an extraction experiment, and the ion contents in the leachate and the extraction solution were calculated, as shown in Table 3. An analysis of the results presented in Table 3 shows that the iron ion content in the leachate from coal gangue was 47.33%, whereas after extraction, the iron ion content in the solution increased dramatically to 99.21%. This indicates that the extraction process effectively separates iron ions and other impurity components from the leachate. Additionally, further experiments were conducted using the high-iron content back-extraction solution to study the process of preparing iron oxide red.

#### 3.3.1. The Influence of pH on the Properties of Iron Oxide Red

**Figure 15:**
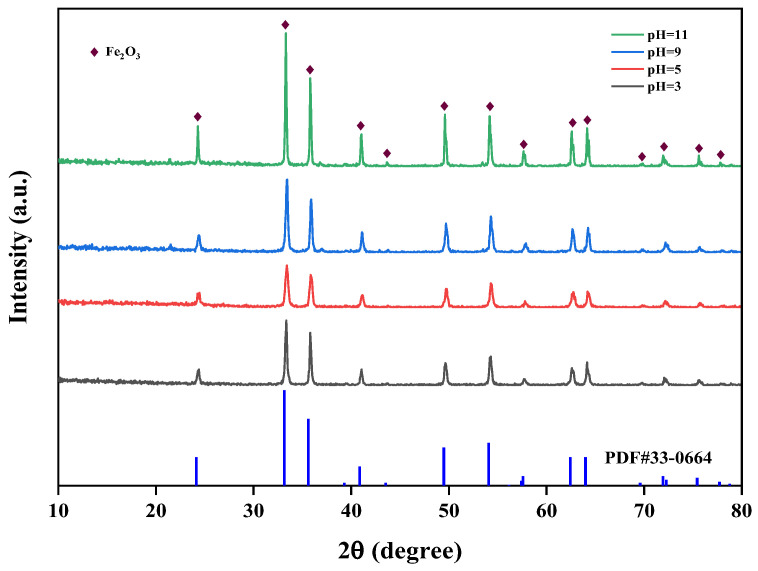
Effect of different hydrothermal pH on iron-leaching rate.

The effect of different pH values (pH = 3, 5, 9, 11) on the crystallinity of iron oxide red was studied at 150 °C and 5 h. The XRD spectra of iron oxide red under different pH conditions are shown in Figure 15. It can be observed from the graph that when the pH is 11, the diffraction peaks of Fe_2_O_3_ are sharper compared to other conditions, indicating that the crystallinity of Fe_2_O_3_ is highest at pH 11. In the hydrothermal synthesis of Fe_2_O_3_, pH is an important influencing factor. A higher pH leads to a faster hydrolysis of Fe^3+^ ions and a higher degree of hydrolysis, resulting in the higher purity of the prepared Fe_2_O_3_.

#### 3.3.2. The Impact of Temperature on the Properties of Iron Oxide Red

**Figure 16:**
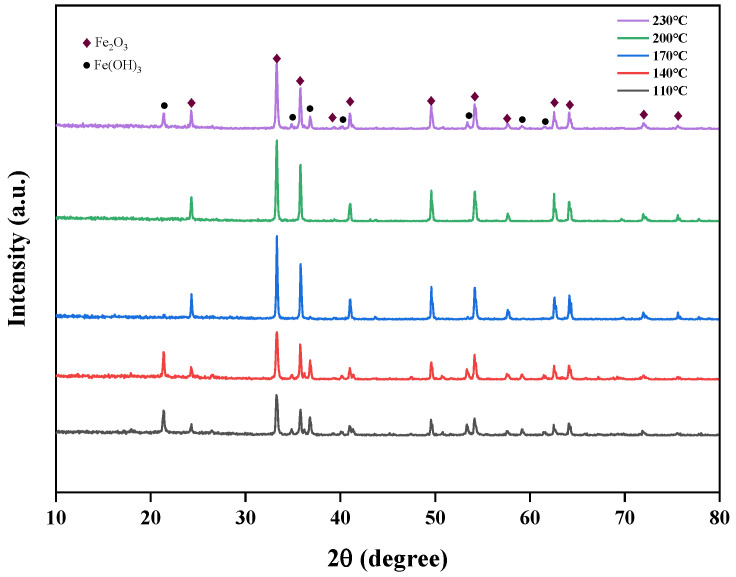
Effect of different hydrothermal temperatures on iron-leaching rate.

The effects of different temperatures (110 °C, 140 °C, 170 °C, 200 °C, 230 °C) on the crystallinity of iron oxide red were studied at pH 11 and a time of 5 h, and the XRD spectra of Fe_2_O_3_ at different hydrothermal temperatures are shown in Figure 16. From the figure, it is evident that at hydrothermal temperatures of 110 °C and 140 °C, the Fe(OH)_3_ phase appears. This is primarily due to the low temperature, where some of the Fe(OH)_3_ phase in the solution has not yet converted to the Fe_2_O_3_ phase. However, at 230 °C, the Fe(OH)_3_ phase reappears, possibly because the high temperature leads to rapid hydrolysis, causing Fe(OH)_3_ to enter the lattice without sufficient conversion. When the hydrothermal temperature is 170 °C and 200 °C, no Fe(OH)_3_ phase is observed, indicating that these temperature conditions are suitable for the transformation from the Fe(OH)_3_ phase to the Fe_2_O_3_ phase. From the perspective of energy conservation, 170 °C was chosen as the optimal hydrothermal temperature for subsequent experiments.

#### 3.3.3. The Effect of Time on the Properties of Iron Oxide Red and the Preparation of Iron Oxide Red under Optimized Process Conditions

**Figure 17:**
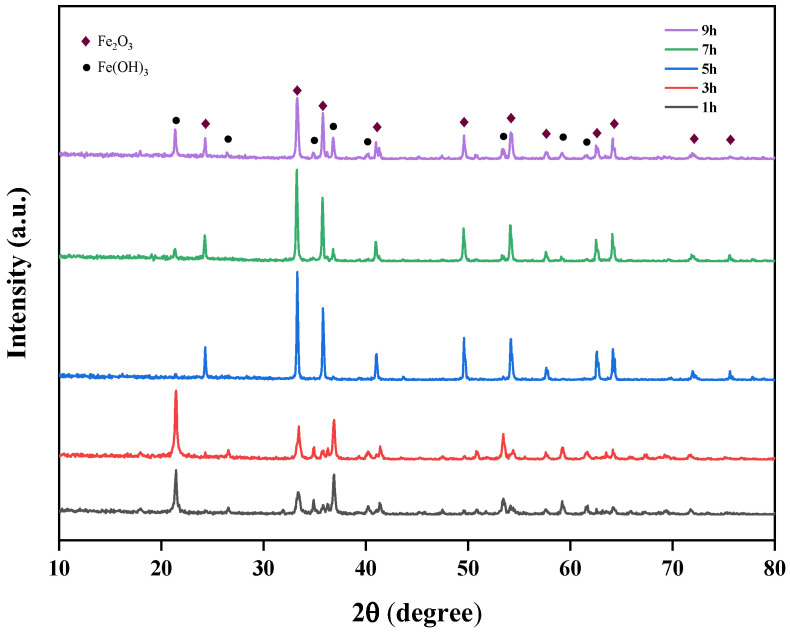
Effect of different hydrothermal times on iron-leaching rate.

At an optimal temperature of 170 °C and pH value of 11, the effect of different durations (1 h, 3 h, 5 h, 7 h, 9 h) on the crystallinity of iron oxide red was investigated. The XRD spectra of iron oxide red under different hydrothermal durations are shown in Figure 17. It can be observed that with an increasing reaction time, both the Fe_2_O_3_ and Fe(OH)_3_ phases are present in the samples. However, as the reaction time extends, the intensity of the Fe(OH)_3_ phase noticeably decreases and its relative content gradually diminishes, indicating that a prolonged reaction time is conducive to purity enhancement. Nevertheless, when the hydrothermal time exceeds 5 h, the peak intensity of the Fe_2_O_3_ phase weakens, and concurrently, the appearance of the Fe(OH)_3_ phase begins. Therefore, the optimal hydrothermal duration for Fe_2_O_3_ phase generation is 5 h.

**Figure 18:**
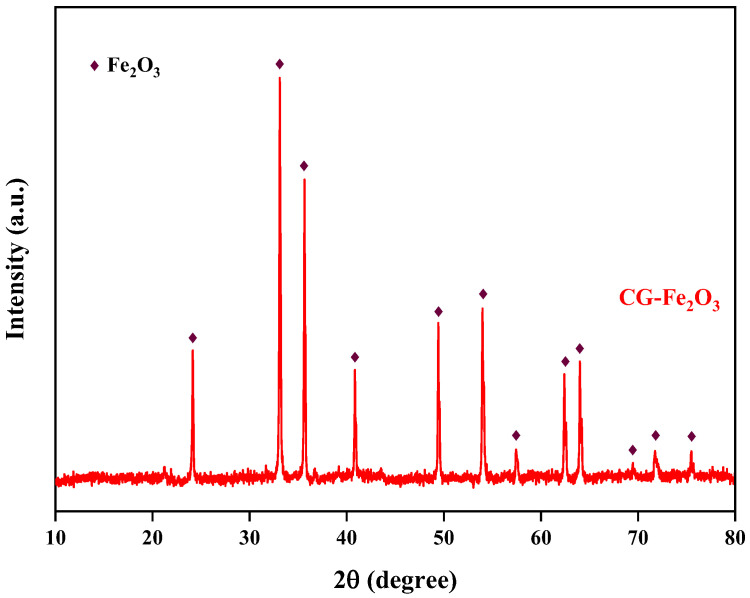
XRD pattern of iron oxide red sample.

**Figure 19:**
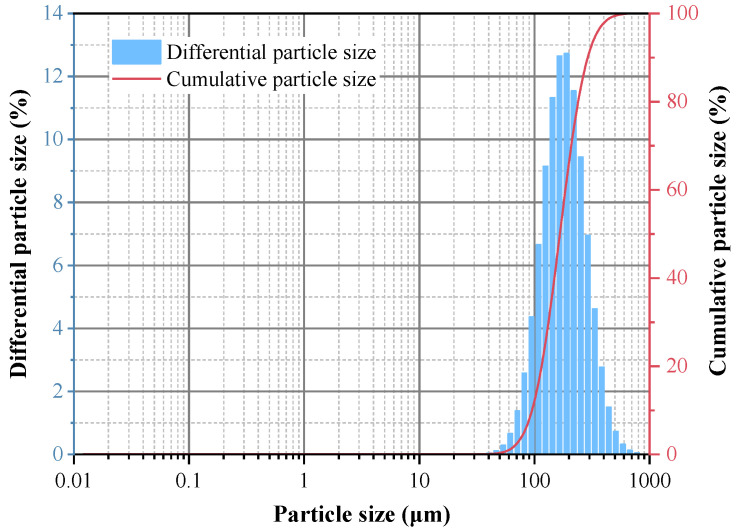
Particle-size distribution of iron oxide red.

The preparation experiment for iron oxide red was carried out under the conditions of optimal reaction pH = 11, reaction temperature 170 °C, and a reaction time of 5 h, and the yield of iron oxide red was 80.07%. The XRD spectrum of the prepared iron oxide is shown in Figure 18. All the diffraction peaks of the sample correspond to the standard card of Fe_2_O_3_ and exhibit sharp peaks [33], indicating a high degree of crystallinity. Additionally, there are no other excess peaks observed in the diffraction peaks, indicating the high purity of the prepared Fe_2_O_3_. The particle-size distribution of iron oxide red is shown in Figure 19, ranging from 25 to 780 μm, with an average particle size of 167.16 μm. The cumulative volume of particles less than 200 μm is 61.95%, and that of particles less than 500 μm is 98.78%. ICP-OES was used for the elemental analysis of the iron oxide red samples. The analysis results are shown in Table 4. The purity of the iron oxide red prepared using coal gangue reached 99.16%, exceeding the purity standard (98.5%) required by the “Iron Oxide for Ferrite YHT4 Standard” (GB/T 24244-2009) [34], making it suitable as a raw material for producing high-performance soft magnetic ferrites.

#### 3.3.4. SEM-EDS Analysis of Iron Oxide Red

**Figure 20:**
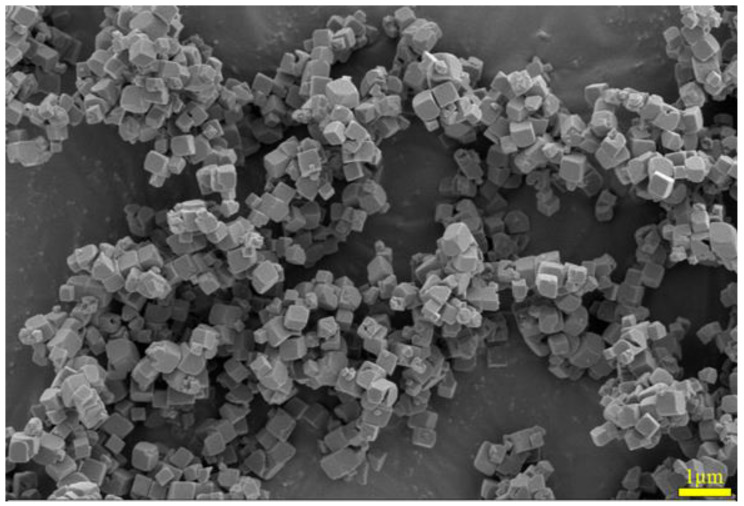
SEM image of iron oxide red sample.

**Figure 21:**
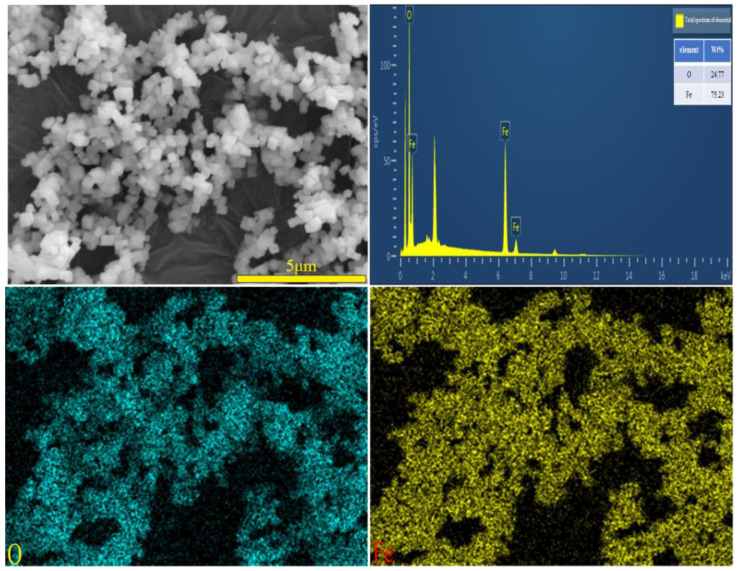
SEM image and EDS mapping analysis of iron oxide red sample.

A SEM image of the iron oxide red sample is shown in Figure 20, depicting particles with a cubic structure and a certain degree of dispersion. The SEM-EDS spectrum of the iron oxide red sample is illustrated in Figure 21, indicating that the particles contain only Fe and O elements. This suggests that the prepared Fe_2_O_3_ has a high purity without any other impurities. Specifically, the Fe element accounts for 75.23% of the total sample mass, while the O element accounts for 24.77%.

### 3.4. Mechanism of Coal Gangue Roasting Activation–Acidic Iron Removal-Extraction–Hydrothermal Preparation of Iron Oxide Red

**Figure 22:**
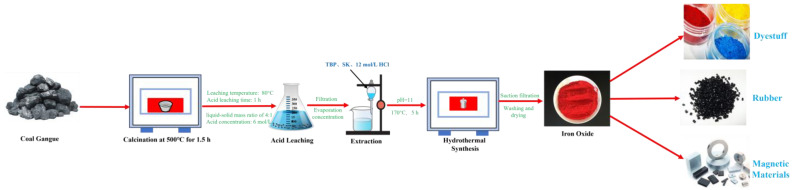
A schematic diagram of the mechanism of coal gangue roasting and activation–acid iron removal–extraction–hydrothermal synthesis of iron oxide red.

The schematic diagram of the process for coal gangue roasting activation, acid leaching iron removal, extraction purification, and the hydrothermal synthesis of Fe_2_O_3_ is shown in Figure 22. During the coal gangue roasting activation stage, some kaolinite and siderite are destroyed [35], and the following reactions occur:

Al_2_Si_2_O_5_(OH)_4_→Al_2_O_3_·2SiO_2_ + 2H_2_O↑ (3)

FeCO_3_ + O_2_→Fe_2_O_3_ + CO_2_↑ (4)

During the acid leaching iron removal stage, the extraction and separation of Fe from impurity ions such as Al, Mg, and Ca take place, with the specific reactions as follows:

Al_2_O_3_·2SiO_2_ + 6HCl→2AlCl_3_ + 2SiO_2_ + 3H_2_O (5)

Fe_2_O_3_ + 6HCl→2FeCl_3_ + 3H_2_O (6)

MgO + 2HCl→MgCl_2_ + H_2_O (7)

CaO + 2HCl→CaCl_2_ + H_2_O (8)

During the extraction purification–hydrothermal synthesis of Fe_2_O_3_ stage, Fe ions are enriched and Al, Mg, and Ca ions are removed. Subsequently, NaOH solution is added, and the mixture is placed into a hydrothermal reactor for synthesis. The reaction equation is as follows:

FeCl_3_ + 3NaOH→Fe(OH)_3_↓+3NaCl (9)

Fe(OH)_3_→Fe_2_O_3_ + 3H_2_O (10)

In summary, the coal gangue undergoes the stages of roasting activation pretreatment, acidic iron removal, extraction purification, and hydrothermal synthesis, leading to the production of high-purity iron oxide red products. This further facilitates the utilization of coal gangue as a solid waste resource.

## 4. Conclusions

(1) The roasting temperature of coal gangue was controlled at 500 °C for 1.5 h, and the iron-leaching rate was 87.64% under the conditions of an optimal iron removal acid concentration of 6 mol/L, an acid leaching temperature of 80 °C, an acid leaching time of 1 h, and a liquid——solid mass ratio of 4:1, which was increased by 20.66% compared with unroasted activation.
(2) A solvent extraction method (TBP-SK-hydrochloric acid system) was employed to extract the leachate, resulting in an iron ion content as high as 99.21% in the extracted solution. Additionally, the extraction agent is economically viable, environmentally friendly, and can be reused.
(3) Iron oxide red products with a yield of 80.07% and a purity of 99.16% were prepared by keeping to an optimal hydrothermal pH = 9, temperature 170 °C and time of 5 h. The characterization analysis using XRD, SEM-EDS, particle-size analysis, and ICP-OES showed that the synthesized iron oxide red showed sharp peaks, the microstructure was similar to the cubic morphology, the average particle size was 167.16 μm, and the purity was 99.16%, which exceeded the purity standard (98.5%) specified in the “Iron Oxide for Ferrite-YHT4” standard (GB/T 24244-2009). This material can serve as a raw material for producing high-performance soft ferrite materials.
(4) The preparation of high-purity iron oxide red through the processes of coal gangue roasting activation pretreatment, acid leaching for iron removal, solvent extraction purification, and hydrothermal synthesis demonstrates significant importance in promoting the high-value utilization of coal gangue resources and alleviating environmental pressure.

## Figures and Tables

**Table 1:** Chemical composition of coal gangue. Unit: %.

Chemical Composition	SiO_2_	Fe_2_O_3_	Al_2_O_3_	CaO	TiO_2_	S	K_2_O	MgO	MnO	P_2_O_5_	LOI
Content	35.5	25.1	14.7	12.9	3.68	2.68	2.56	1.25	0.548	0.399	13.58

**Table 2:** Chemical composition of coal gangue leaching residue. Unit: %.

Chemical Composition	SiO_2_	Fe_2_O_3_	Al_2_O_3_	CaO	TiO_2_	S	K_2_O	MgO	MnO	P_2_O_5_
Content	62.7	4.05	15.5	5.47	6.23	2.71	2.43	0.43	0.34	0.039

**Table 3:** The concentration of each ion in the leaching solution and the back-extraction solution. Unit: mg/L.

Sample	Fe	Al	Ca	Mg
Leaching solution	14,177.90	4183.15	8809.83	2784.12
Back-extraction solution	11,539.51	23.05	48.61	19.17

**Table 4:** Analysis results of iron oxide products composition. Unit: %.

Index	The Content of Each Ingredient													
	Fe_2_O_3_	SiO_2_	CaO	Al_2_O_3_	MnO	TiO_2_	MgO	Na_2_O	K_2_O	P_2_O_5_	NiO	Cr_2_O_3_	CuO	B
Standard Values	≥98.50	≤0.030	≤0.030	≤0.040	≤0.30	≤0.020	≤0.050	≤0.030	≤0.020	≤0.030	≤0.040	≤0.040	≤0.040	—
Measured Values	99.16	0.0276	0.0256	0.0326	0.240	0	0.0411	0.0223	0.0136	0.0289	0.0334	0	0.0310	0.02643

## Data Availability

The data presented in this study are available on request from the corresponding author. The data are not publicly available due to technical or time limitations.
